# Toward the concept of creative planetary health: insights from creative nature-based community practices in the UK

**DOI:** 10.3389/fpubh.2026.1966064

**Published:** 2026-09-30

**Authors:** Linda J. M. Thomson, Manal Aldabbagh, Zoe Moula, Victoria Hume, Helen J. Chatterjee

**Affiliations:** 1 Department of Biosciences, University College London, London, United Kingdom; 2 Department of Arts and Sciences, University College London, London, United Kingdom; 3 Research Division of Care in Long-term Conditions, King's College London, London, United Kingdom; 4 The Culture, Health and Wellbeing Alliance CIC, Barnsley, United Kingdom; 5 Department of Curatorial, Public, and Visual Cultures, University of the Witwatersrand, Braamfontein, Johannesburg, South Africa

**Keywords:** biopsychosocial framework, community programs, creative health, human health and wellbeing, nature-based practices, planetary health, social determinants of health

## Abstract

**Introduction:**

Planetary health recognizes the interdependencies between the health of humans and the state of the environment. The triple planetary crisis of climate change, diversity loss, and pollution impacts unequally on human health depending on environmental and social determinants, exacerbating pre-existing inequalities. Creative nature-based and cultural community practices have the potential to address planetary and human health issues and tackle health inequalities.

**Methods:**

Data triangulation examined convergence and divergence of findings from two qualitative studies of creative nature-based community programs aiming to enhance planetary and human health. Studies using interview and case study designs were conducted by different researchers from the same investigative team.

**Results:**

Thematic analysis identified 11 themes with 23 subthemes for interview data, and eight themes with 25 subthemes for case study data. High levels of convergence were observed between nine themes and 18 subthemes from interview data with six themes and 21 subthemes from case study data. Triangulation and amalgamation of similar themes and subthemes determined six new, more focused themes: Concern about climate change; Connecting with nature; Creativity; Mental health and wellbeing; Mutually supported collaboration; Providing an inclusive environment, and 13 subthemes.

**Conclusions:**

Triangulation increased the study's validity and strengthened its overall credibility. Findings interpreted through a biopsychosocial framework aligned with community, environment, and wellbeing domains. Creativity was represented strongly in both analyses. Findings suggest that creative planetary health practices which promote connection with nature and other people positively impact individual and collective health and wellbeing, and develop care for the environment, thereby supporting the concept of creative planetary health.

## 1 Introduction

Planetary health focuses on characterizing the links between human activities and their effects on planetary and human health (1). A planetary health perspective recognizes that human health and the health of the natural world are one and the same, and to promote human health, humans must protect the health of their planet (1, 2). The United Nations Framework Convention on Climate Change coined the term “triple planetary crisis” to describe the interdependence of climate change, biodiversity loss, and pollution which influence the earth's ecosystems, economies, and societies (3). Human-driven climate change is one of the greatest threats to the planet and affects the determinants of health through extreme weather events such as floods, droughts, heatwaves and wildfires that occur with greater frequency and severity (4). As a “cornerstone of wellbeing” biodiversity provides essential resources and regulating services such as climate moderation, flood mitigation, and nutrient cycling, crucial for maintaining ecological equilibrium [(5), p. 1985]. The emergence of infectious diseases underscores the necessity of preserving biodiversity for global health security (6). Pollution is responsible for nine million deaths globally with air pollution accountable for 1.8 million of these (7).

Concern for the state of the planet can exacerbate existing mental health issues and lead to psychological distress and eco-anxiety (8). Climate anxiety, characterized by intense worry, fear and distress in response to the threats of climate change, can result in significant mental health challenges particularly when individuals feel powerless or overwhelmed by the scale of environmental issues (9). Pre-existing inequities are exacerbated in higher risk populations (10). Climate injustice arises when poorer nations that have contributed far less to climate change are likely to suffer disproportionately from its effects (11). Intergeneration injustice occurs where young people and future generations, who are least responsible for the actions that cause these effects, may experience greater climate and ecological degradation (12). Consequently, issues of justice and human rights run hand in hand with health concerns (3). Climate justice and intergenerational justice are therefore essential in attaining planetary health (2), and acting upon these challenges requires cross-disciplinary and global collaboration (1).

As the formal decision-making body for the United Nations (UN) Framework Convention on Climate Change (UNFCCC), the UN annual climate summit or Conference of Parties (COP) acts to assess progress in combating climate change (3). The 2030 Agenda addressed the five ‘*p*'s of sustainable development goals (people, planet, prosperity, partnership, peace) for a better future (13). International climate convenings have amplified the need to integrate the arts and culture into environmental and planetary health initiatives. UNESCO's meeting on Culture, Heritage, and Climate Change marked a milestone in bridging culture, climate and health (14). Momentum continued at COP27, where the Climate Heritage Network launched the Sharm El-Sheikh Declaration on Culture-based Climate Action, urging governments to enhance collaborations between culture and environment ministries (14). In COP28, the Group of Friends of Culture-Based Climate Action and the Emirates Declaration on Culture-Based Climate Action were also established; an advocacy milestone for integrating culture into global health and climate frameworks; nevertheless, gaps remain between the high-level declarations and formal policy instruments (14).

In addition to tangible health consequences, climate change disrupts both natural and cultural systems that support mental, emotional, and social wellbeing including a sense of safety, identity, place and belonging (15). Sustaining culture is therefore a human rights imperative and requires policies that integrate culture as a critical dimension of climate change and public health (14). Public health challenges cannot be undertaken entirely by healthcare systems, and it is acknowledged that social determinants outside this sector might make a greater contribution to population health outcomes (16); one such approach is creative health. The All-party Parliamentary Group on Arts, Health and Wellbeing (APPGAHW) inquiry report was one of the first publications to use the term “creative health” for a plethora of creative and cultural practices, drawing attention to the policy implications of the role of creativity in health and wellbeing (17). The potential, however, to address planetary and health issues and health inequalities through culture and creativity has not yet been fully realized (18). In addition to cultural and arts-based practices, health outcomes can be improved through creative nature-based interventions (19). Benefits of exposure to natural environments evidence physical, social, cognitive and emotional development for children and young people (20), and connection to nature through the arts is correlated with pro-environmental behavior and wellbeing (21).

In a study assessing the biopsychosocial effects of a museum program involving horticulture, artmaking and collections, adult mental health service users were offered a “green prescription” in an art gallery with adjacent parkland (22). The combination of arts- and nature-based activities was associated with improved psychological wellbeing, decreased social isolation and encouraged self-esteem and a sense of community (22). Analysis of survey data assessing the impact of culture, health- and nature-based engagement on mitigating the adverse effects of COVID-19 public health restrictions showed that engagement with culture, health, and nature-based activities reduced loneliness and improved social connectedness and psychological wellbeing (23). A further study aiming to identify common features of environmentally and socially engaged community programs found major themes of: “Collaboration and partnerships”; “Community health and wellbeing”; “Funding”; and “Mental health” (18), aligning with the Creative Health Quality Framework principles of collaborative, creative, equitable, person-centered, realistic, reflective, safe, and sustainable practices (24). The study indicated a need for inclusive practice, partnership, and longer-term funding to support practitioner wellbeing and sustainable environment-based health interventions instrumental in preventative healthcare (18).

The current study draws upon two biopsychosocial theoretical frameworks through which to interpret its findings. The genomics, environment, nerve, interaction, allostatic regulation, and longevity (GENIAL) model emphasizes connecting with the self, others and the planet to promote wellbeing and longevity (25). The model identifies the role of the vagus nerve in countering the fight and flight response and forming a vital link between physical health and mental wellbeing. Similarly, the tri-dimensional model of behavior includes personal (self-care), social (caring for others) and physical environment (caring for the environment) components and, additionally, highlights that individuals must first recognize and satisfy their own needs to be able to care for others and protect the environment (26).

The current study compared findings from two further studies of practices concerned with enhancing planetary and human health through creative nature-based community programs. The research questions asked to what extent convergence and divergence of findings occurred across these two qualitative studies, whether findings aligned with a biopsychosocial framework, and whether outcomes supported the concept of creative planetary health.

## 2 Methods

### 2.1 Design

Data triangulation was used to examine convergence and divergence of findings from two studies using different designs (interviews and case studies) conducted by different researchers from the same investigative team. The objectives of triangulation were to minimize bias and achieve a comprehensive understanding of enhancing planetary and human health through creative nature-based community programs (27). Both studies used inductive thematic analysis following the six-phase framework (28), and NVivo 14 software to code and analyze the data. The data were analyzed separately and then triangulated.

### 2.2 Participants

Both studies employed purposive sampling, and the interview study additionally used snowball sampling for recruitment. Participants across both studies consisted of UK-based professional adult female creative health practitioners (*n* = 19). While the recruitment methods allowed for mixed-gender samples, the exclusively female cohort potentially reflects gender distributions within the creative practitioner industry. Participants took part in interviews (*n* = 10) (Table 1) or provided case studies (*n* = 9) (Table 2). Interview participants were selected through an informal database of projects collected by the Culture, Health and Wellbeing Alliance (CHWA) and web searches. Case study participants were recruited from CHWA shortlisted annual climate award applicants. For both studies, inclusion criteria consisted of participants facilitating community-based projects addressing planetary and human health that connected to the natural world, were concerned with sustainability, demonstrated an awareness of climate and ecological crises or addressed social justice or eco-anxiety. Exclusion criteria comprised practitioners focused on human health and wellbeing without a planetary health element, and therapies including creative arts therapies as sessions would differ from community-based practices in terms of professional training and guidelines.

**Table 1:** Interview participants [codes] and projects.

Role of participant [code]	Project description	UK region	Target audience(s)
Artist in residence [P01]	Participatory events are organized around outdoor sculptures and optical devices to encourage sensory encounters with nature, understanding woodland ecology and conservation of wildlife.	Scotland	Families with children
Independent artist [P02]	Participatory visual art practices, guided walks and tree-planting as shared experiences of the relationship of humans with landscapes and natural and heritage environments.	North East and North West England	Adults
Independent artist [P03]	Creative practices in natural environments, including dance, movement, free outdoor exercise classes and an annual community walk.	South East England	Local communities
Independent artist [P04]	Eco-theater company questioning where food comes from, creating stories about a future world where natural food has been forgotten, and promoting pro-environmental behaviors and connection to nature.	Wales	Families with children
Artist and founder of organization [P05]	Taking participants out of built environments into local green spaces, working with natural finds and art materials to explore and engage in nature and imagine and record it in creative ways.	East of England	Children and young people and mental health professionals working with families
Artist and director of organization [P06]	Mindfulness photography, regenerative change, and active hope to help participants slow down, observe their surroundings, and connect deeply with nature to address mental health challenges and reframe their perspectives.	South West England and online	Individual adults and organizations
Artist and director of organization [P07]	Performing arts, storytelling, and nature-based activities with artists, educators, and psychologists to educate participants about climate change in an engaging and supportive manner that takes wellbeing into account.	North West England	Children and young people
Founder of organization [P08]	Workshops, nature-based activities, and creative exercises, group discussion and reflective practices that help participants learn from nature and apply these lessons to their lives and work.	East Midlands and online	Individual adults and organizations
Director of organization [P09]	Creative climate change projects focused on raising awareness of climate change through arts and cultural engagement, encouraging pro-environmental behavior and shared knowledge.	Scotland	Local women.
Director of organization [P10]	Community events based on themes such as art, food, gardening, upcycling, mind-body therapies, and outdoor adventure, bringing people together through mutual interests rather than perceived challenges, to share skills and experiences.	Wales	Adults from diverse backgrounds and those needing extra help supported by volunteers

**Table 2:** Case study participants [codes] and projects.

Role of participant [code]	Project description	UK region	Target audience(s)
Community artist and project lead [P11]	Community arts, walking and nature project celebrating and connecting with every day and overlooked aspects of nature to address health issues including addiction, cancer, epilepsy and severe mental illness.	East of England	Adults associated with charity for single homeless people and migrants
Artist and library employee [P12]	Spoken English language sessions exploring topics such as health and global warming, and creative sessions such as using recycled textiles to show rising ocean levels on collaborative world map and individual local maps.	North West England	Migrant communities, many from countries affected by climate change.
Artist and founder [P13]	Live-streamed synchronous events hosting artists from different countries with trees or in woodland to achieve a sense of global kinship, offering experience of natural environments, and reminding people of the unity required for planetary restoration.	Online	Online audiences contributing donations to plant rain forest trees.
Project lead, hospital arts program [P14]	Conversation and open forums on sustainability and the climate using creativity and co-design to reframe thinking from the level of an individual to the collective and share knowledge.	London	All levels of hospital employees from cleaners to executive board members
Creative director [P15]	Video game about climate change co-produced with children and young people during COVID-19 to address mental health issues and gain skills in game design and teamwork.	Yorkshire and the Humber	Children and young people aged 8–20 years recruited for four age-appropriate teams
Founder and director [P16]	Nature, photography and mindfulness to help people slow down, see the world with fresh eyes, reduce anxiety, depression and stress and improve resilience in the face of uncertainty and climate change.	South West England	People from community, museum, education, public and business sectors.
Co-producer/co-presenter [P17]	Podcasts about parenting during a climate emergency discussing the role of carers helping the next generation cope with the climate emergency and ecoanxiety.	London and Northern Ireland	Parents, guardians and carers
Instigator and delivery partner [P18]	Discussions, exhibitions and workshops to support artists, community and ecological groups, schools, colleges and universities to explore their agency within the climate agenda and its relationship with social justice and health.	North West England	Local residents from the most deprived wards and polluted areas, and young people from inner cities
Project collaborator and supporter [P19]	Pop up community spaces that enable residents to come together to develop ideas and projects that tackle the climate and ecological crisis such as planting trees.	London	Residents, families and school children

### 2.3 Materials

Materials comprised two sets of online participant information leaflets and consent forms, an interview protocol with 10 open-ended semi-structured questions (Table 3) and a case study template with four open-ended semi-structured questions (Table 4).

**Table 3:** Interview questions.

Question
1. Can you tell me about your program/activity/work?
2. How does the program engage nature, creativity, and the environment?
3. How does the program address human health and wellbeing?
4. What specific activities, tasks or actions are designed to bring about these outcomes (creativity/environmental/health and wellbeing)?
5. To what extent does your work incorporate sensory (embodied) experiences?
6. How do you evaluate the impact of your work (short or longer term?)? Do you measure any specific human/environmental outcomes?
7. Could you describe the anticipated health and wellbeing outcomes for participants?
8. Are there anticipated environmental outcomes from your work/project?
9. What type of behavior change might you expect to see in participants as a result of your sessions? Do you have any examples?
10. Are you familiar with the terms: planetary health, nature connectedness, and pro-environmental behavior? If yes, to what extent do they influence your work/the project?

**Table 4:** Case study questions.

Question	Word limit
How does your work use creativity/culture to enable the health and wellbeing of people, place and planet, and how does it acknowledge the interconnectedness of people/planetary health? *We are interested in work that joins the dots between health and wellbeing, climate, and creativity/culture*.	200
In what ways have you involved people with relevant lived experiences of environmental challenges and climate impacts? *For example, this may include communities who experience poor air quality or fuel poverty alongside other multiple incidences of deprivation, or young people who experience climate distress or ‘eco-anxiety'*.	150
What are the health or wellbeing impacts and what are the impacts for the environment, or places and planet? *If you have any evaluation statistics, numbers taking part etc., please share them here, along with any stories, quotes or case studies*.	300
Is there anything else you would like to tell us about the project/people involved?	200

### 2.4 Procedure

Ethical approval was obtained for the research. Interview questions were sent out to participants 2 weeks in advance and interviews of 40–60 min duration were conducted one-to-one on a Zoom platform over 14 weeks (May–August 2024). The interview began with an introduction and explanation of the research followed by a brief conversation about participants' backgrounds and professional activities, encouraging participants to share perspectives. Case studies were drawn from submissions for CHWA's Climate Awards recognizing projects leading by example in acknowledging the connections between climate, health and creativity or culture; shortlisted applicants had the opportunity to showcase their work nationally through promotion on the CHWA bulletin, website and social media channels. Shortlisted applicants were subsequently approached (August–September 2025) and asked if they would like their submission to be included in research exploring creative planetary health practices and, if so, they were asked to read the participant information leaflet and sign the consent form in MS Forms.

## 3 Results

Thematic analysis identified 11 themes with 23 subthemes for interview data (Figure 1) and 8 themes with 25 subthemes for case study data (Figure 2). Themes and subthemes with examples of representative quotations are outlined for each analysis (quotations are shown with participant codes in squared brackets, listed in Tables 1, 2). Triangulation of interview and case study data follows the analyses.

**Figure 1:**
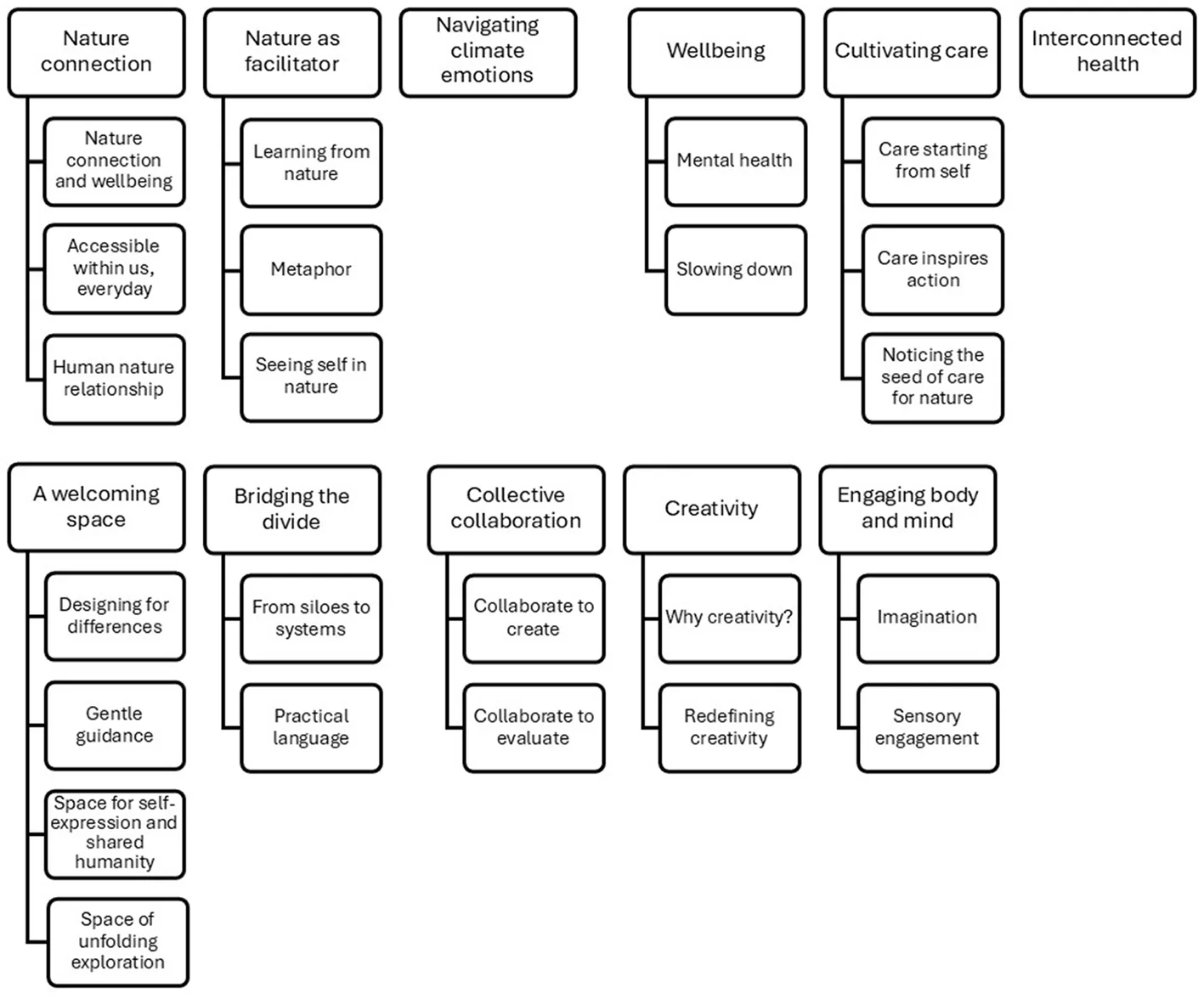
Themes and subthemes from the interview analysis.

**Figure 2:**
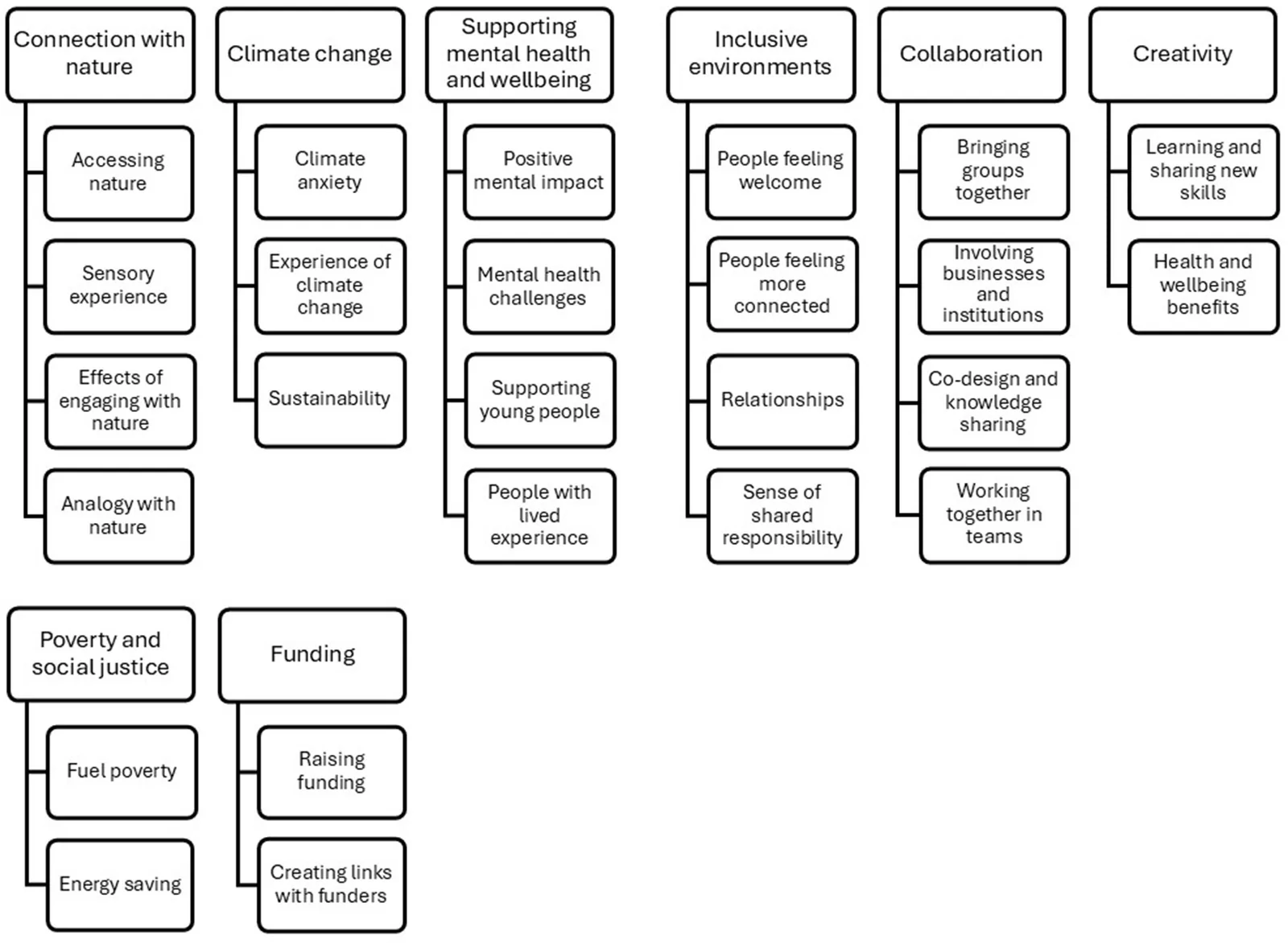
Themes and subthemes from case study analysis.

### 3.1 Interview themes

#### 3.1.1 Nature connection

Connecting with nature was a fundamental principle among practitioners. The subtheme “Nature connection and wellbeing” reflects the strong link between the natural environment and mental health and wellbeing. An arts practitioner facilitating nature walks explained that “people always come out of the walk being uplifted, people say comments like my mood totally changed” [P02]. The subtheme “Accessible within us, everyday” emphasizes nature's presence in every aspect of life. A workshop facilitator referred to a student's comment that “we are nature, there is a sense of being part of something” [P05]. The subtheme “Human nature relationship” highlights a reciprocal relationship with nature; as a practitioner stated, “we don't extract and we give back as much as we possibly can” [P06].

#### 3.1.2 Nature as facilitator

Participants saw that the natural environment acted as a facilitator actively contributing to activities and outcomes. For the subtheme “Learning from nature”, a practitioner acknowledged, “nature has a huge amount of wisdom to offer us and that we can learn from” [P06]. A facilitator explained that “its nature that's doing the talking; we feel that we're facilitators between us and the rest of the living world and it's actually the rest of the living world that's sharing the wisdom” [P08]. For the subtheme “Metaphor”, nature-based metaphors improved accessibility and facilitated discussion. For the subtheme “Seeing self in nature”, participants were encouraged to observe similarities, as an arts practitioner described “I get them to look at the palm of their hand and draw the patterns they can see… and then we go for a walk and see all the patterns, the same as the flow in the river, or it's the same as the roots in the tree” [P03].

#### 3.1.3 Navigating climate emotions

Facilitators fostered a sense of agency and empowerment to help participants deal with challenging climate emotions, as a facilitator explained “it's giving people permission to explore the possibility of hope” [P07]. Another facilitator emphasized the importance of balancing the need to engage children with the realities of the climate and biodiversity crises while also prioritizing their wellbeing, “we do see a lot of ecoanxiety and climate anxiety as well, and that shows up in lots of different forms… we then support people to acknowledge that as a process of grief… when those emotional stages have been gone through, people can then move forward” [P08].

#### 3.1.4 Wellbeing

As an integral part of the research, practitioners discussed the extent to which projects addressed human wellbeing. For the subtheme, “Mental health”, practitioners emphasized that “environmental connection is so closely linked to wellbeing, and particularly people's mental health” [P01] and “the whole point of it was to help people to improve their mental health and their wellbeing” [P06]. For the subtheme of “Slowing down”, an artist explained that it was something they were “trying to encourage” [P01]. Slowing down was related to mindfulness by an artist who clarified that it was “about the present moment and… we're looking and we're seeing with gratitude, with compassion, with non-judgment, we're letting go of stuff” [P06].

#### 3.1.5 Cultivating care

The importance of cultivating care was highlighted for both the person and the environment. The subtheme “Care starting from self” was expressed by a practitioner stating, “you have to start with mechanisms and structures about how you care for yourself, and then, how you care for people beyond that” [P03]. The subtheme “Care inspires action” was based on the notion that appreciating nature encourages people to take action to protect and preserve it, as a facilitator explained “it's about connecting people to nature and the environment… the likelihood is that will change their behavior in that they're not going to drop the litter because they respect and enjoy it” [P10]. The subtheme “Noticing the seed of care for nature” was illustrated by facilitators who explained that “once nature speaks to people, they will become the champions of nature and its care in the future” [P05], and “it just comes so naturally with kids; when they're playing in a place that they love, they care for it” [P09].

#### 3.1.6 Interconnected health

Practitioner responses concerned the interconnection of planetary and human health and how it informed their practices. In response to the question about familiarity with planetary health, nature connectedness, and pro-environmental behavior, a facilitator expressed “I am very aware of it, I think they do influence our work” [P09] and an independent artist stated, “they're just both the same thing as far as I see it, the health of our planet and the health of us as humans and we're completely interconnected” [P04]. Permaculture based on natural ecosystems was a conceptual framework that participants used to inform their projects, as illustrated by “when I learned about permaculture in relation to gardening, I realized that can be very much transferred over to communities and people” [P10] and “permaculture as a process of connection to the whole… in the sense of this natural planet, this system that we're all part of.” [P03].

#### 3.1.7 A welcoming space

The subtheme “Designing for differences” recognizes diversity in engaging with nature and providing a welcoming environment, as an artist explained “I'm anticipating in some senses that most of those reactions will be positive, but I also have to acknowledge that some may be negative” [P01]. The subtheme of “Gentle guidance” involved structured activity to foster curiosity without imposing rigid guidelines; “a theme that helps to set the tone for the project” [P02]. Storytelling was a means of communicating concepts; an artist described “creating stories around how we are connected to nature” [P04] and a facilitator spoke about “a magical storyline where it's quite fantastical because that age group is still willing to enter into that realm of fantasy” [P09]. For the subtheme “Space for self-expression and shared humanity”, practitioners created a “non-judgmental space” where people could “find their spot and take time and space to engage in the nature around them” [P05]. The subtheme “Space of unfolding exploration” reflected a fluid process of “leading people into that reconnection with other living beings and then seeing what emerges naturally” [P08].

#### 3.1.8 Bridging the divide

The need to bridge the divide and align academic and medical research with real-world concerns appears crucial. The subtheme “From siloes to systems” illustrates the cost to society of compartmentalizing humans, particularly in education, highlighted by “we're still so set in boxes with the curriculum like the subjects, and there's supposed to be lots of cross-curricular” [P09]. The subtheme “Practical language” reflects the necessity to use accessible language rather than jargon or complex terminology, as expressed by “I don't know if I like the term ‘pro-environmental behavior'… I just think it's jargonistic” [P10] and “the use of language is really important in a community context… to help people along their journey of understanding” [P07].

#### 3.1.9 Collective collaboration

The subtheme “Collaborate to create” reflects the need to involve diverse professionals to consider a project's processes and outcomes, illustrated by a facilitator who explained “we had a theater director… we also had environmental scientists, environmental consultants in the room… and we also had education experts” [P09]. The subtheme “Collaborate to evaluate” recognized the need for a robust evaluation process to assess a project's impact despite most projects lacking essential resources such as money, expertise and time, as expressed by a practitioner, “it would be amazing to work with an academic who would have that interest [P02]”.

#### 3.1.10 Creativity

The role of creativity was a key influence in the design and delivery of projects. The subtheme “Why creativity?” examines responses as to why participants involved creative processes in their practices. An artist pointed out that a “creative space… enables the self-reflection work that's needed to go on without it being articulated or verbalized” [P07], while a director of an arts organization explained it was about “the process of change, of reframing, and of creating something new” [P06]. The subtheme “Redefining creativity” suggests that it is lessons learnt through the process of “taking yourself on a creative and immersive journey into nature” [P05] that are important, rather than outcomes or goals.

#### 3.1.11 Engaging body and mind

Two approaches were evidenced, one engaging the mind through imagination and the other, the body through sensory experience. The subtheme, “Imagination”, envisages a positive future on both individual and environmental levels and considers actions that could be taken. An artist explained that they asked participants to imagine their local area and then write a story “about what that looks like in 50 years in a world where we are living connected to nature… to essentially plan what they could do with their garden or local community space” [P04]. The subtheme of “Sensory engagement” was derived from a direct question about the extent to which participants incorporated sensory experiences, as a practitioner described “participants stop, listen and get their hands dirty, they often work sitting on the ground or in among plants or trees, the separation between ourselves and nature thins” [P05].

### 3.2 Case study themes

#### 3.2.1 Connection with nature

A core element of all projects was connection with nature. The subtheme “Accessing nature” shows that practitioners were open to the idea that “nature could be accessed with simple tools, such as observation, different ways to walk, taking a slower pace and artmaking” [P11]. The subtheme “Sensory experience” was illustrated by a participant “transported into a global forest with all senses activated” [P13]. The subtheme “Effects of engaging with nature” involved celebrating “the nourishing experience of engaging in nature” that helped their participants “feel more conscious and live in the moment” [P11]. The subtheme “Analogy with nature” referred to comparisons made with everyday life such as the seasons. The same artist related life stories to self-seeded plants around a town which became a “symbol of our respect for all lives and journeys” [P11].

#### 3.2.2 Climate change

For the subtheme “Climate anxiety” a facilitator explained that engagement helped people “deal with their fear, grief and increasingly eco/climate anxiety and encourages action and connection” [P16]. Activities included a film project with farmers about the importance of wildflower meadows and a digital art installation about the environmental significance of rivers. The subtheme “Experience of climate change” involved participants and volunteers with experience of climate impacts such as flooding in Bangladesh. A series of podcasts asking, “How do you parent in a climate emergency?” consulted some of the world's leading climate thinkers on “how to rip up “parenting” and piece it back together to fit our planetary reality” [P17]. The subtheme “Sustainability” reflected the intentions of practitioners to introduce “vocabulary related to sustainability and environmentalism” [P12] and use recycled and sustainable materials in their projects.

#### 3.2.3 Supporting mental health and wellbeing

Communities were involved in projects to support their mental health and wellbeing; as an artist explained “participants found the sessions to be a calming activity which had a positive effect on their wellbeing” [P12]. The subtheme “Positive mental impact” reflected the observation that “engaging had a calming and restorative effect” which made participants feel “grounded” [P11]. For the subtheme “Mental health challenges”, the same arts facilitator explained that participants had experienced “addiction, cancer, epilepsy and severe mental illness” and had “accessed nature to support their health” [P11]. The subtheme “Supporting young people” illustrates the effects of interventions to increase resilience and improve educational and social outcomes, as a young person expressed “my confidence and resilience has grown enormously, I'm now back at college studying photography, focusing my work on the impact of climate change” [P16]. The subtheme “People with lived experience” included facilitation of activities by those who had undergone homelessness, migration and mental illness.

#### 3.2.4 Inclusive environments

For inclusivity, a project focused on “building relationships and celebrating voices often on the margins with people and groups of all ages, cultures, and abilities” [P15]. The subtheme “People feeling welcome” reflected the need for “a welcoming environment in which people could engage with cultural and creative activities” [P12]. One facilitator described how a disused café was “converted into a welcoming space accessible to all including families and school children” [P19]. The subtheme “People feeling more connected” arose “as a result of meeting others in the space” [P19]. For the subtheme “Relationships”, “Mapping relationships as we move forward” [P19] strengthened the bond between groups tackling the climate crisis. For the subtheme “Sense of shared responsibility”, a practitioner expressed that it was important to “pursue mutual relationships” [P14].

#### 3.2.5 Collaboration

An artist grew their group project by “sharing ideas and tasks in a mutually supportive collaboration” [P13]. The subtheme “Bringing groups together” was typified by “partnering and supporting 44 community and ecological groups, schools, further and higher education, and artists to explore their agency within the climate agenda” [P18]. The subtheme “Involving businesses and institutions” was included because practitioners identified the necessity to involve businesses and institutions to ensure “projects are taken forward” [P19]. The subtheme, “Co-design and knowledge sharing”, involved exchanging information, with one participant suggesting “a climate change knowledge wall” [P19]. For the subtheme “Working together in teams”, projects agreed that “no single person has the answer… reinforcing the need to work together in teams” [P14].

#### 3.2.6 Creativity

A practitioner explained how they supported the emotional development of young people on “an amazing creative journey from inception to completion of a video game made for general release” [P15]. For the subtheme “Learning and sharing new skills”, a facilitator encouraged “learning by doing” [P19], and an arts practitioner described the value of “participation through co-design and sharing knowledge as part of embedded learning and reflective practice” [P14]. An artist provided “hands-on media literacy training for other artists offering informal learning in the use of widely available digital technologies” [P13]. For the subtheme “Health and wellbeing benefits”, after years of antidepressants, one of this artist's participants experiencing mental health issues chose “recovery by natural means after years of anti-depressant medication, leading to the promotion of creativity as key to emotional wellness” [P13].

#### 3.2.7 Poverty and social justice

Several projects involved poverty and social justice; a facilitator described partnering and supporting groups to explore “their agency within the climate agenda and its relationship to social justice and health and wellbeing” [P18]. For the subtheme “Fuel poverty”, residents in one of the most deprived wards described “experiencing fuel poverty due to increased prices as an effect of climate change” and that they were also adversely affected “rising food prices” [P12]. For the subtheme “Energy saving”, several projects addressed poverty directly such as by providing “a guide to simple energy saving” [p18].

#### 3.2.8 Funding

One practitioner described making two series of podcasts about the climate crisis as a “labor of love with no funding and little resources” [P17], a typical position for most community projects. Practitioner questions about small grant applications were echoed in the subtheme “Raising funding”. A arts facilitator explained that taking part in arts activities should be “cost-free to ensure diversity and equality of participation” [P13], but this also meant they were unable to pay facilitators; with funding they would be able to offer fees to artists. The subtheme “Creating links with funders” was seen as one solution, though practitioners questioned how they might take the proposal forward and “create links with industry/institutions” [P19].

### 3.3 Triangulation of interview and case study data

There was a high level of convergence of the two analyses that shared commonalities from nine themes (82%) and 18 subthemes (78%) of the interview data with six themes (75%) and 21 subthemes (84%) of the case study data. Similar themes and subthemes across the analyses were amalgamated and renamed (Table 5). Six new, more focused themes: Connecting with nature; Concern about climate change; Mental health and wellbeing; Providing an inclusive environment; Mutually supported collaboration; and Creativity, and 13 new subthemes amalgamated from both data sources (Figure 3) served to increase the validity and credibility of the findings. In terms of the level of divergence, two themes (18%) and five subthemes (22%) of the interview data and two themes (25%) and four subthemes (16%) of the case study data showed less overlap with existing themes or the six new themes formed through amalgamation.

**Table 5:** New themes and subthemes amalgamated from interview and case study thematic analyses.

New themes	Themes		New subthemes	Themes	Subthemes	
	Interview analysis	Case study analysis		Interview analysis	Interview analysis	Case study analysis
Connecting with nature	Nature connection	Connection with nature	Accessing nature in daily life		Accessible within us, everyday	Accessing nature
			Engaging the senses		Sensory engagement	Sensory experience
			Analogy, metaphor and seeing oneself in nature		Metaphor	Analogy with nature
					Seeing self in nature	
Concern about climate change	Navigating climate emotions	Climate change	Navigating climate emotions/eco-anxiety	Navigating climate emotions		Climate anxiety
			Pro-environmental behavior	Interconnected health		Sustainability
Mental health and wellbeing	Wellbeing	Supporting mental health and wellbeing	Wellbeing and positive impact		Nature connection and wellbeing	Effects of engaging with nature
						Positive mental impact
			Addressing mental health		Mental health	Mental health challenges
Providing an inclusive environment	A welcoming space	Inclusive environments	Feeling welcome in a space	A welcoming space		People feeling welcome
			Connection and sharing with other people		Space for self-expression and shared humanity	People feeling more connected
					Space of unfolding exploration	Sense of shared responsibility
Mutually supported collaboration	Collective collaboration	Collaboration	Relationships between individuals and organizations	Interconnected health	Collaborate to create	Bringing groups together
						Co-design and knowledge sharing
						Relationships
			Involving businesses and institutions		Collaborate to evaluate	Involving businesses and institutions
Creativity	Creativity	Creativity	Lessons learnt in the process of creating		Why creativity?	Learning and sharing new skills
					Imagination	
			Benefits of creativity		Redefining creativity	Health and wellbeing benefits

**Figure 3:**
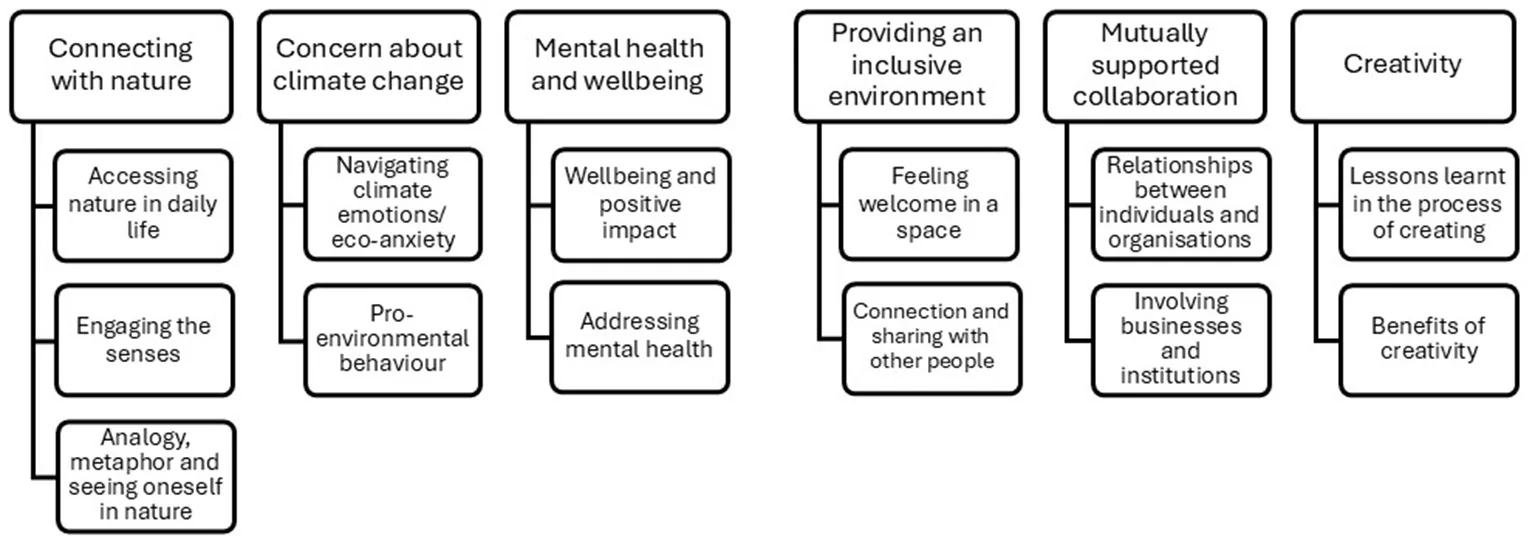
Triangulation of themes and subthemes from the interview and case study analyses.

The interview analysis included divergent themes of “Cultivating care” and “Bridging the divide”, whereas the case study analysis included divergent themes of “Poverty and social justice” and “Funding”. “Cultivating care” included care for the planet, that bore some similarities with the case study subtheme “Sustainability”. “Bridging the divide” referred to the need for cross-disciplinary research and education addressed partly by the case study subthemes of “Bringing groups together” and “Working together in teams”, though these did not include education. The subtheme “Poverty and social justice” was not covered by the interview analysis, and the subtheme “Funding” was only touched upon in the interview analysis by the subtheme “Collaborate to evaluate” that reflected upon the need for funding to conduct robust evaluation.

## 4 Discussion

The research questions asked to what extent convergence and divergence of findings occurred across two qualitative studies of creative health practices concerned with enhancing planetary and human health through nature-based community programs, whether findings aligned with a biopsychosocial framework, and whether outcomes supported the concept of creative planetary health. Three quarters or more of the combined themes and subthemes converged with divergence at a quarter or less. Aligning with a previous study (18), triangulation showed that a common thread across both analyses was the connection of humans with nature and, linked to that, climate emotions and pro-environmental behavior. Interconnection within nature is one of the five domains of the Planetary Health Education Framework (1), and findings concur with the proposal that a sole focus on human health is no longer sustainable and should be considered within the context of planetary health (4).

In keeping with the concept of “creative health” (17), outcomes from the current study supported the concept of “creative planetary health” encapsulating the interconnectedness between creative health and planetary health. The concept aligns with the definition of creative planetary health that refers to “care delivered through a planetary health lens by creative health practitioners using their expertise to tackle the environmental, commercial and social determinants of health” [(29), p. 1]. Furthermore, the intersection of the natural world with creativity and health has the capacity to enhance human and planetary health through inspiring creativity and improving wellbeing (30). As it is argued that collective imagination is essential to finding solutions to planetary health crises, creativity might be vital in envisioning a more harmonious future for planetary and human health (31). To this end, it is important to acknowledge the traditions and knowledge systems of Indigenous Peoples which innately recognize culture, arts and creativity as core ingredients of health and wellbeing (32).

Although acknowledging the range of climate emotions appears essential to drive effective climate action, considering mental health and wellbeing and implementing ethical safeguards in climate-related initiatives is a necessity (8). Activities that involve connecting with nature can have positive impacts on wellbeing, yet climate anxiety characterized by a feeling of being overwhelmed by the scale of environmental issues and manifest as helplessness, grief and anger, can also occur (9). Both analyses in the current study converged on the theme, mental health and wellbeing, recognizing the positive impact of nature-based projects and how they might offer resilience to life's challenges and mitigate existing mental health issues (21). A balanced approach is recommended where practitioners acknowledge the severity of climate change while emphasizing the need for collaboration to promote sustained pre-environmental behavior (9).

Current findings align with several domains of the GENIAL biopsychosocial framework (25). New themes amalgamated from both analyses included connecting with nature and concern about climate change corresponding to GENIAL's environment domain; mental health and wellbeing corresponding to the wellbeing domain; and connection and sharing with other people and mutually supported collaboration identifying with the community domain. Although the new themes did not explicitly include the individual domain, subthemes concerning engaging the senses and seeing oneself in nature shared a resemblance.

Surprisingly, the theme of creativity, represented strongly in both analyses and a core element of practice, was not an explicit domain of the GENIAL model. For the tri-dimensional model of behavior (26), the social and physical environment components aligned with new themes of mutually supported collaboration, connecting with nature and concern about climate change. The self-care component was implicit in mental health and wellbeing and explicitly stated in the interview analysis as care starting from self, a subtheme of cultivating care. The premise that individuals must recognize and satisfy their own needs before caring for others and protecting the environment, was touched upon in the subtheme of care starting from self in the interview analysis but not present in the case study analysis or the amalgamated themes.

### 4.1 Limitations

In conducting triangulation of qualitative data, the researchers recognize that they were never removed from the research process and acknowledge that their understanding of the data may have predisposed them to certain conclusions. The research was conducted from an interpretivist point of view, acknowledging that reality can be multiple and subjective. The role of triangulation helped to shape interpretations, mitigated researcher bias, and contributed to the overall rigor of the study. Triangulation required reflexivity and critical thinking on the part of the researchers exploring different facets of phenomena and how meanings were constructed through various lenses. The two researchers involved in the analyses acknowledge their reflexivity and input into the studies and to reduce possible bias, two independent researchers were involved in reviewing the process. Owing to these limitations, consensus about conclusions regarding amalgamation of the findings should be treated with relative caution.

## 5 Conclusions

This study highlights the transformative potential of creative and nature-based projects for fostering a healthier and more sustainable world. Healthcare and creative health practitioners should collaborate with experts, other organizations and government bodies to actively engage in advocacy and policy related to the interdependence of planetary and human health. In conducting nature-based projects, practitioners need to offer a safe, non-judgmental space that respects individual differences and encourages exploration, particularly in nature. Practitioners should also be aware of climate/eco-anxiety and support participants in navigating climate emotions constructively. Furthermore, practitioners should minimize their negative impact on the planet and commit to sustainable practices and materials, using their expertise to address the triple planetary crisis. Findings suggest that creative planetary health practices which promote connection with nature and other people positively impact individual and collective mental health and wellbeing, and develop care for the environment, thereby supporting the concept of creative planetary health.

## Data Availability

The raw data supporting the conclusions of this article will be made available by the authors, without undue reservation.
